# Unraveling Genomic and Pathogenic Features of *Aeromonas ichthyocola* sp. nov., *Aeromonas mytilicola* sp. nov., and *Aeromonas mytilicola* subsp. *aquatica* subsp. nov.

**DOI:** 10.3390/ani15070948

**Published:** 2025-03-26

**Authors:** Nihed Ajmi, Muhammed Duman, Batuhan Coskun, Ceren Esen, Oner Sonmez, Gorkem Tasci, Orkide Coskuner-Weber, Hilal Ay, Digdem Yoyen-Ermis, Artun Yibar, Andrew P. Desbois, Izzet Burcin Saticioglu

**Affiliations:** 1 Department of Aquatic Animal Diseases, Graduate School of Health Science, Bursa Uludag University, Bursa 16059, Turkey; nihed.ajmi.95@gmail.com (N.A.); grkmrocker12@gmail.com (G.T.); 2 Department of Aquatic Animal Diseases, Faculty of Veterinary Medicine, Bursa Uludag University, Bursa 16059, Turkey; mduman@uludag.edu.tr; 3 Institute of Aquaculture, University of Stirling, Stirling FK9 4LA, UK; batuhan.coskun@stir.ac.uk (B.C.); andrew.desbois@stir.ac.uk (A.P.D.); 4 Department of Immunology, Faculty of Medicine, Bursa Uludag University, Bursa 16059, Turkey; ccereneesen@gmail.com (C.E.); dyoyenermis@uludag.edu.tr (D.Y.-E.); 5 Department of Biochemistry, School of Veterinary Medicine, Bursa Uludag University, Bursa 16059, Turkey; onersonmez@uludag.edu.tr; 6 Molecular Biotechnology, Turkish-German University, Sahinkaya Caddesi, No. 106, Beykoz, Istanbul 34820, Turkey; weber@tau.edu.tr; 7 Department of Molecular Biology and Genetics, Faculty of Arts and Science, Yildiz Technical University, Istanbul 34220, Turkey; hilal.ay@yildiz.edu.tr; 8 Department of Food Hygiene and Technology, Faculty of Veterinary Medicine, Bursa Uludag University, Bursa 16059, Turkey; artunyibar@uludag.edu.tr

**Keywords:** *Aeromonas*, *Galleria mellonella*, genome analysis, taxonomy

## Abstract

The Gram-negative genus *Aeromonas* contains diverse bacterial species that are prevalent in aquatic environments. This present study describes three novel *Aeromonas* strains: *A. ichthyocola* sp. nov. A-5^T^ and *A. mytilicola* subsp. *aquatica* subsp. nov. A-8^T^ isolated from rainbow trout (*Oncorhynchus mykiss*), and *A. mytilicola* sp. nov. A-7^T^ isolated from mussels (*Mytilus galloprovincialis*), respectively. Genomic analyses revealed that strains A-5^T^ and A-7^T^ shared the highest *16S rRNA* gene sequence similarity with *A. rivipollensis* P2G1^T^ (99.7% and 99.8%, respectively), while strain A-8^T^ exhibited 99.7% identity with *A. media* RM^T^. Together with morphological, physiological, and biochemical data, genome-based analyses provided additional evidence for species differentiation. Digital DNA–DNA hybridization (dDDH; 56.8−65.9%) and average nucleotide identity (ANI; 94.2–95.7%) values fell below the species delineation thresholds, confirming that these isolates represent distinct taxa. Pathogenicity assays using greater wax moth (*Galleria mellonella*) larvae demonstrated strain-specific virulence profiles. Further genomic analyses identified biosynthetic gene clusters for nonribosomal peptides (NRPs) and ribosomally synthesized and post-translationally modified peptides (RiPPs), which often have roles in secondary metabolite production. Ecological analyses, based on genomic comparisons and metagenomic database searches, revealed the adaptability of the strains to diverse habitats, including freshwater, wastewater, and activated sludge. Based on the genetic and phenotypic data, the novel taxa *Aeromonas ichthyocola* sp. nov. A-5ᵀ (LMG 33534ᵀ = DSM 117488ᵀ), *Aeromonas mytilicola* sp. nov. A-7ᵀ (LMG 33536ᵀ = DSM 117490ᵀ), and *Aeromonas mytilicola* subsp. *aquatica* subsp. nov. A-8ᵀ (LMG 33537ᵀ = DSM 117493ᵀ) are proposed.

## Simple Summary

This research aimed to describe three newly identified bacterial strains, *Aeromonas ichthyocola* A-5^T^ and *Aeromonas mytilicola* subsp. *aquatica* A-8^T^ isolated from rainbow trout (*Oncorhynchus mykiss*), and *Aeromonas mytilicola* A-7^T^ isolated from mussels (*Mytilus galloprovincialis*). These strains are proposed to belong to the genus *Aeromonas*, which includes bacteria commonly found in aquatic environments. Phenotypic and genotypic analyses were performed to show that these bacteria form two new species and one subspecies. Pathogenicity tests conducted in insect larvae (*Galleria mellonella*) revealed differences in virulence. This study contributes to our understanding of bacterial diversity in aquatic ecosystems and offers insights into their potential roles in aquaculture and biotechnology.

## 1. Introduction

The genus *Aeromonas* includes Gram-negative, oxidase-positive, and facultative anaerobic bacteria that are prevalent in aquatic environments, food products, and aquatic organisms. *Aeromonas* originates from the Greek terms *aer* (air) and *monas* (unit or cell), historically referencing their ability to grow in both aerobic and anaerobic conditions and reflecting their metabolic versatility. These bacteria are of significant interest due to their ecological roles and clinical relevance, particularly as opportunistic pathogens in both aquatic and human health contexts [1].

The pathogenicity of *Aeromonas* spp. has been a focus for extensive investigation due to its ability to infect diverse hosts. In aquaculture, *Aeromonas* species are major opportunistic pathogens responsible for diseases such as hemorrhagic septicemia, furunculosis, and motile *Aeromonas* septicemia in fish hosts such as salmonids, tilapia [2,3,4], and catfish [5], often triggered by environmental stressors such as poor water quality, overcrowding, or inadequate handling practices [6,7]. These infections result in substantial economic losses and pose significant challenges to aquaculture sustainability. Beyond fish, *Aeromonas* infections have been reported in other aquatic organisms, including amphibians, reptiles, and crustaceans, where systemic infections are often observed under conditions where the hosts are in a stressed state [8].

In humans, several *Aeromonas* species are opportunistic pathogens that can cause a spectrum of infections, including gastroenteritis, wound infections, and, in severe cases, septicemia [9,10,11]. The pathogenic potential of these bacteria is driven by a complex array of virulence factors, including extracellular enzymes, toxins, and secretion systems, which enable colonization, immune evasion, and host tissue damage [12,13].

The greater wax moth (*Galleria mellonella*) larva model is widely used as an alternative *in vivo* platform to evaluate bacterial virulence, and it can distinguish strain-specific differences, including for bacterial pathogens of fish [14,15] and human [16,17]. In particular, this model has proven invaluable in advancing our understanding of the complex host-pathogen interactions mediated by *Aeromonas* spp. [18].

This present study aimed to characterize three novel *Aeromonas* isolates obtained from rainbow trout (*Oncorhynchus mykiss*) and mussels (*Mytilus galloprovincialis*) using polyphasic methods and determine their pathogenic potential by experimental infection of *G. mellonella* larvae. The integration of data from phenotypic assays, genomic analyses, and pathogenicity experiments allowed us to deepen our understanding of the pathogenic potential of these three *Aeromonas* strains.

## 2. Materials and Methods

### 2.1. Sampling

Strains A-5^T^ and A-8^T^ were collected from rainbow trout (*O. mykiss*) sampled from commercial fish farms located in Adapazarı (2015) and Duzce (2021), respectively. The fish samples varied in weight, with A-5^T^ isolated from a 120-g trout and A-8^T^ from a 1 kg trout. Sampling followed established protocols for diagnosing fish diseases and complied with international animal welfare standards. The health status of the sampled organisms was assessed based on visible signs of disease. The rainbow trout exhibited non-specific disease symptoms, including darkening in color and skin ulceration; however, no pathognomonic lesion indicative of an *Aeromonas* infection was observed. Before sampling, each fish was euthanized with MS-222 (Merck, Darmstadt, Germany, Cat. No. E10521) to ensure humane handling. Kidney and spleen samples were aseptically collected and inoculated onto trypticase soy agar (TSA; Merck, Darmstadt, Germany) and blood agar (BA; Merck, Darmstadt, Germany). As optimal growth conditions of psychrophilic *Aeromonas* is typically 22–28 °C under laboratory conditions [19], the inoculated agar plates were incubated at 28 °C for 24–48 h to facilitate bacterial growth.

Strain A-7^T^ was obtained from raw mussels (*M. galloprovincialis*) collected in Istanbul Province in May 2023. The mussels, with an average weight of 15.26 g and an average length of 7.19 cm, showed no visible signs of disease. Internal shell contents from the mussels were homogenized, and a 10 g portion of the mixture from each batch was transferred into sterile stomacher bags (Seward Medical, London, UK). Following this, 90 mL of sterile phosphate-buffered saline (PBS; Oxoid, ThermoFisher, Milano, Italy) was added, and the mixture was thoroughly homogenized. Ten-fold serial dilutions were prepared and plated on TSA. The agar plates were incubated aerobically at 28 °C for 24–48 h to allow colony development.

All isolates were cultured in tryptic soy broth (TSB) at 28 °C for 48 h to permit long-term preservation. The cultures were supplemented with 20% glycerol and stored at −80 °C to ensure viability for future analyses.

### 2.2. Morphological, Biochemical, and Physiological Tests

Colony characteristics were examined on TSA plates, and Gram staining was performed using a commercial kit (bioMérieux, Marcy l’Étoile, France). Cellular morphology was analyzed with a transmission electron microscope (JEM 1220, JEOL, Tokyo, Japan) after applying negative staining with 2% uranyl acetate on carbon-coated copper grids. Oxidase activity was assessed using Bactident Oxidase test strips (Merck, Darmstadt, Germany, Cat. No. 1.00181)), while catalase activity was confirmed by adding 3% hydrogen peroxide and observing immediate bubble formation. Sulfide Indole Motility (SIM) medium (Merck, Darmstadt, Germany) was utilized to evaluate motility, indole production, and hydrogen sulfide (H_2_S) generation. The oxidative and fermentative metabolism of the strains was investigated using Hugh and Leifson’s O/F medium. To determine growth characteristics, isolates were cultured on various types of media, including nutrient agar (NA; Merck, Darmstadt, Germany), R2A agar (BD Difco, Franklin Lakes, NJ, USA), bile aesculin agar (BAA; Merck), MacConkey agar (MC; Merck, Darmstadt, Germany), seawater agar (SwA; HiMedia, Mumbai, India), brain heart infusion agar (BHIA; Merck, Darmstadt, Germany), TSA, thiosulfate-citrate-bile salts-sucrose agar (TCBS; Oxoid, Basingstoke, UK), marine agar (MA; BD Difco), and 5% sheep blood agar (BA; Merck, Darmstadt, Germany). Anaerobic growth was evaluated using the AnaeroPack system (Mitsubishi Gas Chemical Co., Inc., Tokyo, Japan) for up to two weeks. The temperature tolerance and optimal growth temperature were determined for each isolate on TSA following incubation at 0, 4, 10, 15, 20, 25, 30, 35, 37, 42, and 45 °C. Enzyme activities were analyzed on a variety of substrates. DNA degradation was tested using DNase agar (HiMedia, Mumbai, India), while lipase activity was evaluated on R2A agar supplemented with 1% (*w*/*v*) Tween 20 or Tween 80. Hydrolysis of starch, gelatin, and casein was analyzed using starch agar (1%, *w*/*v*), gelatin agar (1%, *w*/*v*), and skimmed milk agar (3%, *w*/*v*), respectively, with R2A as the base medium. Hydrolysis of L-tyrosine was examined using L-tyrosine agar (0.5%, *w*/*v*), and bile aesculin agar was employed to evaluate esculin hydrolysis. Further biochemical profiling was performed using API 20NE and API 20E test strips (bioMérieux, Marcy l’Étoile, France) and BIOLOG GENIII MicroPlates (Biolog, Hayward, CA, USA), with incubation at 28 °C for 24–48 h.

### 2.3. Assessment of Biofilm Formation and Antibiotic Susceptibility

The antibiotic susceptibility of the strains was evaluated using the disc diffusion method, following CLSI VET03/VET 04-S2 guidelines for bacteria from aquatic animals [20]. The tested antibiotics included florfenicol (30 µg), doxycycline (30 µg), oxytetracycline (30 µg), flumequine (30 µg), amoxicillin (25 µg), erythromycin (15 µg), trimethoprim/sulfamethoxazole (1.25/23.75 µg), and enrofloxacin (5 µg). Mueller Hinton agar (MHA) plates were inoculated with bacterial suspensions standardized to a 0.5 McFarland turbidity. Antibiotic discs were placed on the plates and incubated at 28 °C for 24–28 h. The diameters of inhibition zones were measured in millimeters, with strains showing no zones considered to be resistant. *Escherichia coli* ATCC 25922 was used as a quality control strain, with inhibition zone measurements verified against CLSI VET03/VET 04-S2 guidelines.

The ability of the strains to form biofilms was determined using the crystal violet staining method [21,22]. Briefly, cultures grown in TSB at 28 °C for 24 h were stained, and biofilm formation was quantified by measuring the optical density (OD) at 600 nm. Biofilm formation was categorized as absent, low, moderate, or high, and all experiments were performed in triplicate based on previously described methods [21,22].

### 2.4. Gene Seqeuncing Analysis Based on 16S rRNA

The genomic DNA of the three strains was extracted using the NucleoSpin Microbial DNA kit (Macherey–Nagel, Düren, Germany) following the manufacturer’s instructions. The DNA concentrations were measured using Qubit 4™ fluorometer using the dsDNA HS kit (Thermo Fisher Scientific, Waltham, MA, USA). The *16S rRNA* gene was amplified and sequenced using the universal primers 27F and 1492R under the following PCR conditions: an initial denaturation at 94 °C for 30 s, followed by 30 cycles of 94 °C for 30 s, 55 °C for 30 s, and 68 °C for 1 min. A final extension step was performed at 68 °C for 5 min. PCR products were purified and sequenced using Sanger sequencing [23,24]. The similarity index of the *16S rRNA* genes from each strain was determined by comparison to the GenBank ‘16S ribosomal RNA sequences (Bacteria and Archaea)’ database.

The *16S rRNA* gene sequences of *Aeromonas* type strains were obtained from the List of Prokaryotic Names with Standing in Nomenclature (LPSN; https://lpsn.dsmz.de/genus/Aeromonas, accessed on 13 October 2024) and aligned using the ClustalW algorithm within MEGA X software (version 10.2.2). *E. coli* ATCC 11229 was included as an outgroup to root the phylogenetic tree. Phylogenetic trees were reconstructed using three methods: neighbor-joining (NJ), maximum-parsimony (MP), and maximum-likelihood (ML), all performed in MEGA X. The NJ and ML algorithms employed Kimura’s two-parameter evolutionary distance model to generate distance matrices, while for the ML tree, a discrete gamma distribution (+G) was applied to account for rate variation across sites, and some sites were modeled as evolutionarily invariable (+I). The NJ tree similarly utilized a gamma distribution to model rate variation. The MP tree was constructed using the tree–bisection–regrafting (TBR) method, starting with 10 initial trees. The reliability of the resulting tree topologies was evaluated through bootstrap analyses with 1000 replicates, ensuring robust confidence values for each node.

### 2.5. Multilocus Phylogenetic Analysis (MLPA)

MLPA analysis using multiple housekeeping genes was employed to overcome the limitations of *16S rRNA* gene sequencing in resolving closely related *Aeromonas* species and thus to achieve higher phylogenetic resolution. Seven housekeeping genes (*gyrB*, *gyrA*, *recA*, *rpoD*, *dnaJ*, *dnaX*, and *atpD*) were extracted from the genomes of the *Aeromonas* type strains. All sequences were aligned using the MAFFT algorithm for accurate alignment consistency across the datasets. Phylogenetic trees were constructed using the ML and NJ methods in MEGA X. The Jukes–Cantor model was applied to calculate nucleotide substitution rates for both methods. For the ML trees, a discrete gamma distribution (+G) was employed to model rate variation among sites, along with an invariable sites parameter (+I). Bootstrap analysis with 1000 replicates was performed to evaluate the stability and confidence of tree topologies [19].

### 2.6. Genome Sequencing and Analysis

A hybrid sequencing approach was employed for genomic analysis, combining short-read and long-read sequencing methods. Short-read libraries were prepared using the Nextera XT DNA Library Preparation Kit and sequenced on the Illumina NovaSeq 6000 platform in 2 × 150-bp paired-end (PE) mode with a 1000-cycle HiSeq reagent kit. Long-read libraries were generated from 400 ng of genomic DNA using the Oxford Nanopore Ligation Sequencing Kit (SQK-NBD114-24; Oxford Nanopore Technologies, Oxford, UK) according to the PromethION protocol provided by the manufacturer, without fragmentation. These long-read libraries were loaded onto a PromethION Flow Cell and sequenced for 24 h using the P2 Solo sequencer (ONT). The high quality reads were assembled into contigs through de novo assembly using the Unicycler hybrid assembly pipeline v.0.4.6 [25]. The draft genome sequence data were submitted to GenBank, and contigs over 1000 bp were annotated using the NCBI Prokaryotic Genome Annotation pipeline (PGAP) [26].

To perform the digital DNA–DNA hybridization analyses together with a comprehensive phylogenomic analysis, the type strain genome server pipeline (TYGS, https://tygs.dsmz.de/, accessed on 20 December 2024) was used to compare the genomes of strains A-5^T^, A-7^T^, and A-8^T^ with the genomes of the type strains deposited in the DSMZ database [27]. The average nucleotide identity values based on BLASTN (ANIb) and MUMMER (ANIm) algorithms were calculated for our strains together with their phylogenetic neighbors to determine overall genome relatedness indices using the JSpeciesWS software (v4.2.3) tool available at https://jspecies.ribohost.com/jspeciesws/, accessed on 20 December 2024 [28].

The Virulence Factor Database (VFDB) (https://www.mgc.ac.cn/VFs/main.htm, accessed on 30 December 2024) was used for the prediction of virulence factors [29]. The PHASTEST (https://phaster.ca/, accessed on 2 January 2025) [30] server was used to identify genomic islands for prophages. Additionally, analysis of antibiotic resistance genes was performed with the Comprehensive Antibiotic Resistance Database (CARD) tool (https://card.mcmaster.ca/analyze, accessed on 30 December 2024) [31], while a search for bioactive secondary metabolite gene clusters was conducted using the AntiSMASH database (v7.0) (https://antismash.secondarymetabolites.org/, accessed on 30 December 2024) [32], and PathogenFinder (https://cge.food.dtu.dk/services/PathogenFinder/, accessed on 30 December 2024) was used to predict pathogenicity towards human hosts [33].

### 2.7. Ecological Distribution

Ecological distribution analysis was performed by aligning the *16S rRNA* gene sequences of the strains with a database of 19,000 amplicon datasets. This was followed by genomic comparisons with 49,094 high-quality metagenome-assembled genomes (MAGs) using the Protologger web tool (https://www.protologger.de/, accessed on 30 December 2024) [34]. Additionally, the presence and distribution of the strains in various metagenomic samples, including those from reptilian hosts, were evaluated using the Integrated Microbial Next Generation Sequencing (IMNGS) platform (https://www.imngs.org/, accessed on 30 December 2024) [35].

### 2.8. Pathogenicity in G. mellonella

*G. mellonella* larvae, weighing approximately 220–300 mg, were obtained from UK Waxworms Ltd. (Sheffield, UK). Upon receipt, larvae were stored in the dark at 4 °C for up to two weeks. Bacterial suspensions of *Aeromonas* strains were prepared and adjusted to desired OD600 using PBS. Ten larvae per condition were injected with 10 μL of bacterial suspension into the final left proleg using a sterile Hamilton syringe (Sigma-Aldrich Ltd., St. Louis, MO, USA). Larvae were incubated at 22 °C and 37 °C for up to 120 h. Three control groups were included: one group injected with 10 μL of sterile PBS; an unmanipulated group to account for background mortality; whilst *Vibrio anguillarum* ATCC 43305, a known pathogen in this host [14], was included as a positive control to benchmark virulence. The bacterial concentrations used for infection trials were ca. 1 × 10⁸, 1 × 10⁷, 5 × 10⁶, and 1 × 10⁵ CFU (colony forming unit)/mL, unless otherwise stated. The concentrations were estimated based on a previously established OD600-to-CFU/mL correlation. Serial dilution and plating confirmed that OD600 ≈ 1.0 corresponded to approximately 1 × 10⁹ CFU/mL [36]. Larval survival was monitored at 24 h intervals by observing movement after a gentle touch with a sterile inoculation loop [14]. The experiments were performed in triplicates.

## 3. Results

### 3.1. Phenotypic Characteristics

The strains were Gram-negative, aerobic, and exhibited rod-shaped morphology (Figure S1). On TSA, the colonies were cream-colored, convex, and circular, with smooth surfaces and entire margins. Oxidase and catalase activities were detected for each strain. Table 1 outlines the phenotypic characteristics distinguishing the three strains from their closest phylogenetic counterparts. A comprehensive list of phenotypic traits for each of these proposed type strains is provided in Table S1, while detailed descriptions are presented in Table 2.

### 3.2. Biofilm Formation and Antibiotic Susceptibility

When evaluated using the crystal violet staining method, biofilm formation was not observed for any of the strains under our incubation conditions, indicating an absence of adherence and aggregation capacity typically associated with biofilm-forming bacteria.

Antibiotic breakpoints are not available in the CLSI and EUCAST databases for *Aeromonas* spp., with the exception of cefepime, ceftazidime, aztreonam, ciprofloxacin, levofloxacin, and trimethoprim–sulfamethoxazole. Thus, the antimicrobial susceptibility values of the strains were not evaluated as susceptible or resistant according to the EUCAST and CLSI cut-off values. In this present study, the strains were defined as resistant when there was the absence of a measurable growth inhibition zone (0 mm). All three strains exhibited resistance to amoxicillin (AML). Strain A-5^T^ displayed a broader antibiotic resistance profile, showing resistance also to sulfamethoxazole–trimethoprim (SXT), oxytetracycline (OT), florfenicol (FFC), and doxycycline (DO) (Table S1).

### 3.3. Phylogenetic Analysis

Nearly full-length *16S rRNA* gene sequences were obtained for strains A-5^T^ (1363 bp), A-7^T^ (1357 bp), and A-8^T^ (1358 bp) and subjected to comparative analysis. Strains A-5^T^ and A-7^T^ showed the highest pairwise identity with *A. rivipollensis* P2G1^T^ (NR_144574.1), at 99.7% and 99.8%, respectively, while strain A-8^T^ exhibited 99.7% identity with *A. media* RM (NR_036911.2). Comparisons among the isolates revealed 99.9% identity between strains A-5^T^ and A-7^T^, 99.4% between strains A-5^T^ and A-8^T^, and 99.3% between strains A-7^T^ and A-8^T^ (Figure S2).

### 3.4. Multilocus Phylogenetic Analysis (MLPA)

**Figure 1:**
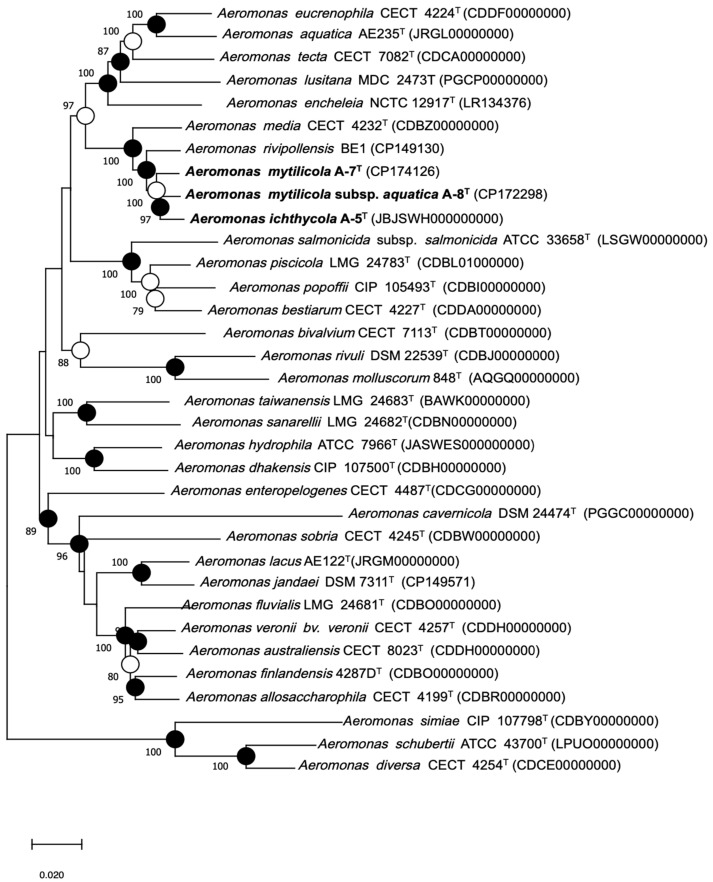
Multilocus phylogenetic analysis (MLPA) was performed to construct a phylogenetic tree from concatenated sequences of seven housekeeping genes (*gyrB*, *rpoB*, *rpoD*, *dnaJ*, *recA*, *gyrA*, and *atpD*), which illustrates the relationships among all described *Aeromonas* species, including type strains, reference strains, and selected isolates. Novel strains described in this study—A-5ᵀ, A-7ᵀ, and A-8ᵀ—are highlighted in bold. Filled circles indicate branch nodes recovered by ML, NJ, and MP phylogenetic trees; open circles indicate that corresponding nodes were also recovered in either ML or MP algorithm. Numbers at nodes indicate bootstrap values (percentage of 1000 replicates). Bar represents 0.02 estimated nucleotide substitutions per site.

To improve species-level resolution, MLPA was conducted using concatenated sequences of seven housekeeping genes (*gyrB*, *rpoB*, *rpoD*, *dnaJ*, *recA*, *gyrA*, and *atpD*). Phylogenetic trees were built using both the ML and NJ methods, with bootstrap values > 70% considered to provide strong support (Figure 1). Strains A-5^T^, A-7^T^, and A-8^T^ form distinct clades and are closely related to *A. rivipollensis* BE1. Strains A-5^T^ and A-8^T^ clustered closely, while strain A-7^T^ was located on a different branch within the same group. Despite their similarity to *A. media* CECT 4232^T^, the unique phylogenetic placement of the three new strains suggests they could represent new variants within the genus *Aeromonas*.

### 3.5. Genome Analysis

**Figure 2:**
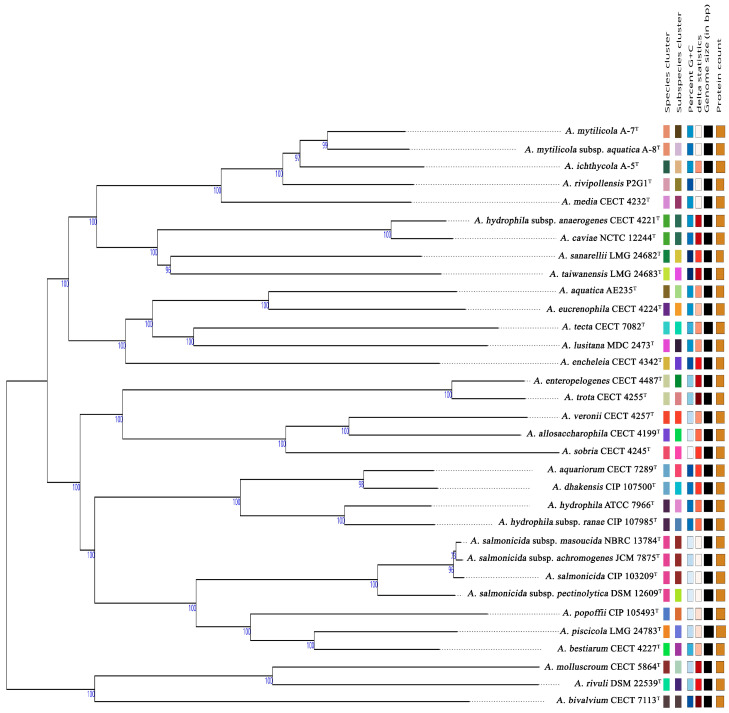
Delineation of *Aeromonas* strains based on GBDP phylogenetic analysis retrieved from the TYGS platform. The tree was inferred with FastME 2.1.6.1 using GBDP distances calculated from genome sequences, and branch lengths are scaled according to the GBDP distance formula d5. Numbers above branches represent GBDP pseudo-bootstrap support values (>60%) from 100 replications. The tree was rooted at the midpoint. Right-hand side columns provide additional genomic information: species and subspecies clusters are represented with different colors for classification purposes. Color gradients in GC content and delta statistics (δ values) columns are for visual comparison. Genome size (bp) and protein count columns are visually represented by bars, where longer bars correspond to larger genomes or higher protein counts.

**Figure 3:**
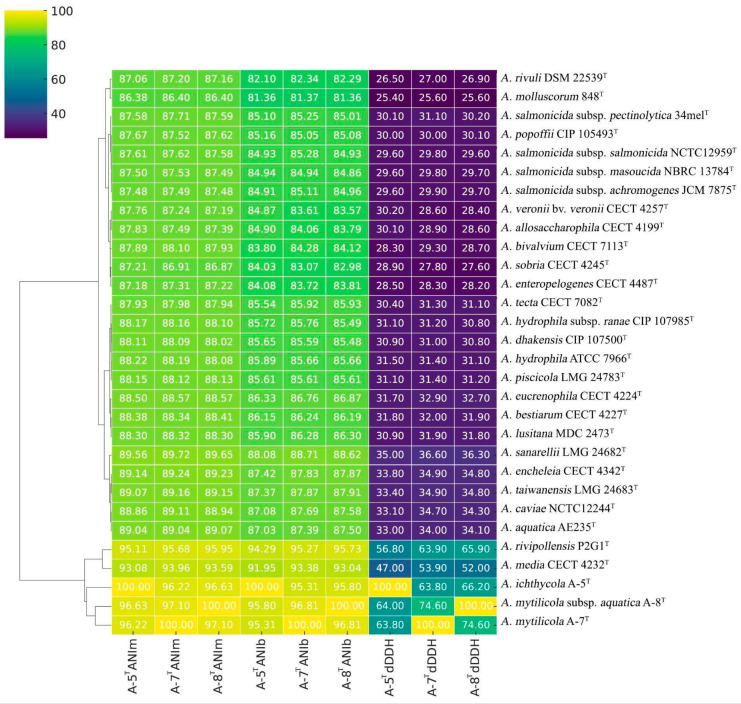
Heatmap representation of the genetic similarity among various *Aeromonas* species, including the newly described strains (A-5^T^, A-7^T^, and A-8^T^). Heatmap illustrates pairwise similarity percentages, with a color gradient transitioning from light shades (lower similarity) to darker shades (higher similarity), providing a visual representation of genetic relationships. Numerical values indicate calculated similarity percentages for quantitative interpretation.

The genomes of strains A-5^T^, A-7^T^, and A-8^T^ have been submitted to the NCBI GenBank under accession numbers JBJSWH000000000, CP174126-CP174129, and CP172298, respectively. The genome sizes of these strains range between 4.6−4.7 Mb, with GC contents varying from 61.14−61.40% (Table S2). Genome-based phylogenetic analyses were conducted using the type strain genome server (TYGS). The phylogenomic tree demonstrated close relationships between strains A-5^T^, A-7^T^, and A-8^T^, with an average branch support value of 98.8% (Figure 2). Digital DNA–DNA hybridization (dDDH) values between strains A-5^T^, A-7^T^, and A-8^T^ and other *Aeromonas* type strains were consistently below the species delineation threshold of 70%, supporting their classification as potential novel species or distinct subspecies within the genus (Figure 3). Comparative genomic analyses with *A. rivipollensis* P2G1^T^ revealed ANI values calculated using the BLAST algorithm of 94.29%, 95.29%, and 95.73% for strains A-5^T^, A-7^T^, and A-8^T^, respectively, and corresponding values obtained using the MUMmer algorithm of 95.11%, 95.68%, and 95.95%. The dDDH values for these strains ranged from 56.8−65.9%, which are all below the 70% threshold for species delineation. A comparative analysis of dDDH and ANI values indicated that strain A-5^T^ represents a distinct species, while strains A-7^T^, and A-8^T^ belong to the same species. The dDDH value between A-7^T^, and A-8^T^ (74.60%) falls within the 70–80% range, suggesting a subspecies-level relationship, with A-8^T^ likely representing a subspecies of A-7^T^. In contrast, the dDDH values between A-5^T^ and both A-7^T^ (66.20%) and A-8^T^ (63.80%) remain below 70%, further confirming A-5^T^ to be a separate species. These findings are further supported by high ANI values (>95%) among the three strains, indicating their close genomic relationship within the *Aeromonas* genus.

A comprehensive genomic analysis using the CARD database identified 11 antibiotic resistance genes across the genomes of strains A-5^T^, A-7^T^, and A-8^T^ (Table S3). These genes confer resistance to multiple classes of antibiotics, including cephalosporins, penams, carbapenems, sulfonamides, and aminoglycosides. Notably, strain A-8^T^ was found to harbor the *TRU-1* gene, encoding a beta-lactamase enzyme associated with resistance to beta-lactam antibiotics. Strain A-7^T^ carried the *OXA-917* gene, conferring resistance to carbapenems, while strain A-5^T^ contained the *sul2* gene, which mediates resistance to sulfonamides.

In addition to antibiotic resistance genes, 89 virulence-related genes were identified across the genomes of strains A-5^T^, A-7^T^, and A-8^T^ (Table S4). These genes were associated with motility, adherence, and toxin production, which are key factors that may contribute to the pathogenic potential of these isolates. Notably, strain A-8^T^ harbored genes encoding components of the lateral flagella, potentially enhancing its colonization and motility capabilities in aquatic and host environments. Both A-7^T^ and A-8^T^ shared genes associated with the mannose-sensitive hemagglutinin (Msh) pilus. Additionally, the strains contained genes for type IV pili and polar flagella.

The prophage analysis identified distinct genetic elements within the genomes of strains A-5^T^, A-7^T^, and A-8^T^ (Table S5). Strain A-8^T^ harbored a 44 kb intact prophage associated with PHAGE_Klebsi_ST15_OXA48phi14.1 (NC_049454), encoding 57 proteins with a GC content of 59.14%. In contrast, strain A-7^T^ exhibited two prophage regions: a 35.6 kb questionable region linked to PHAGE_Escher_Lys12581Vzw (NC_049917), which encodes 33 proteins and has a GC content of 60.70%, and a 40.1 kb intact prophage region associated with PHAGE_Entero_Mu (NC_000929), encoding 47 proteins with a GC content of 58.70%. Strain A-5^T^ contained a 37.7 kb intact prophage region linked to PHAGE_Aeromo_phiO18P (NC_009542), consisting of 44 proteins with a GC content of 58.34%.

The analysis of biosynthetic gene clusters (BGCs) revealed diverse secondary metabolite biosynthesis potential among the three strains (Table S6). Strain A-7^T^ contained five BGCs, including three RiPP-like clusters, one NRP-metallophore/NRPS cluster with 100% similarity to amonabactin P, and one hserlactone cluster. Similarly, strain A-5^T^ harbored four BGCs, including two RiPP-like clusters, one NRP-metallophore/NRPS cluster identical to amonabactin P, and one hserlactone cluster. Strain A-8^T^ exhibited a similar BGC profile, with four clusters, including two RiPP-like clusters, an NRP-metallophore/NRPS cluster identical to amonabactin P, and a hserlactone cluster. The presence of the amonabactin-associated NRPS cluster in all three strains suggests a potential adaptation mechanism for iron acquisition, which may play a role in survival in iron-limited environments.

The pathogenic potential of strains A-5^T^, A-7^T^, and A-8^T^ was evaluated *in silico* using PathogenFinder, which predicts pathogenicity based on genomic similarity to known pathogens. All three strains were classified as non-human pathogens, with pathogenicity probability scores of 0.461 (A-5^T^), 0.469 (A-7^T^), and 0.457 (A-8^T^), all below the 0.5 threshold indicative of human pathogenicity.

Strains A-5^T^ and A-8^T^ lack plasmids, while strain A-7^T^ harbors three plasmids, encoding a diverse array of 17 genes. These include the Type II toxin-antitoxin system RelE/ParE family toxin, a helix–turn–helix domain-containing protein, a CopG family ribbon–helix–helix protein, MobV and MobQ family relaxases, a plasmid replication protein (RepB), a TraY domain-containing protein, a mobilization protein, a partial replication initiation protein, and several hypothetical proteins (Table S7).

### 3.6. Ecological Distribution and Habitat Preferences

The Protologger spider plots and IMNGS platform analyses *in silico* provided a comprehensive understanding of the predicted ecological distribution and habitat preferences of strains A-5^T^, A-7^T^, and A-8^T^ across 19 diverse environments. The Protologger results identified activated sludge to be the primary habitat, exhibiting the highest detection frequencies, followed by freshwater and wastewater. This reflects the adaptability of the strains to both engineered and natural aquatic ecosystems. Moderate prevalence was also observed in soil and rhizosphere habitats, indicating their potential capacity to thrive in terrestrial environments (Figure S3). The IMNGS analysis, which evaluated over 19,000 amplicon datasets, offered additional insights into the ecological versatility of the strains. Strain A-5^T^ demonstrated the highest prevalence in gut metagenomes, with over 3.3 million strain-level matches (≥99% identity), followed by common carp (*Cyprinus carpio*) and freshwater habitats. Strain A-7^T^ exhibited a similar pattern to A-5^T^, showing significant representation in gut-associated and freshwater environments. In contrast, strain A-8^T^ displayed distinct preferences for biofilm habitats, with 892,000 matches, alongside moderate detection in gut (1.3 million matches) and *C. carpio* metagenomes (1.4 million matches). The gut metagenomes analyzed in IMNGS include samples from a diverse range of hosts, including vertebrates such as humans, fish, and amphibians, as well as invertebrates like leeches. Collectively, these results highlight the predicted ecological flexibility of the three strains and their likely ability to colonize a variety of niches, with a strong affinity for aquatic, engineered, and host-associated habitats (Table S8).

### 3.7. Pathogenicity of Strains in G. mellonella

**Figure 4:**
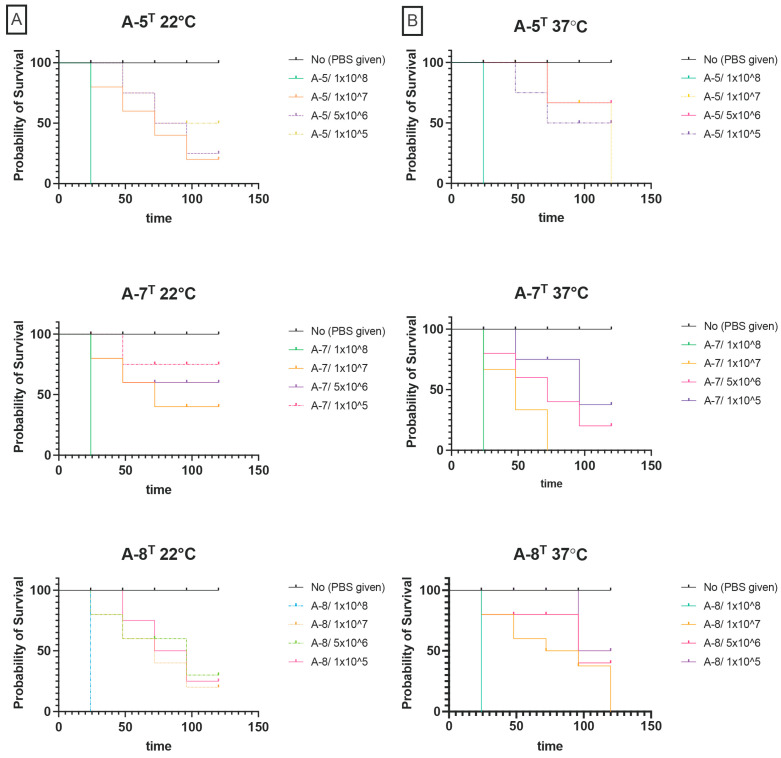
(**A**) Kaplan–Meier survival curves of *Galleria mellonella* infected with strains A-5^T^, A-7^T^, and A-8^T^ at 22 °C. (**B**) Kaplan–Meier survival curves of *G. mellonella* infected with strains A-5^T^, A-7^T^, and A-8^T^ at 37 °C.

The virulence of strains A-5^T^, A-7^T^, and A-8^T^ was evaluated in *G. mellonella* larvae. At 22 °C, strain A-5^T^ caused complete mortality at 1 × 10^8^ CFU/mL within 24 h. At 1 × 10^7^ CFU/mL, survival declined to 80% at 24 h, 40% at 48 h, and 10% at 72 h. At lower concentrations (5 × 10^6^ and 1 × 10^5^ CFU/mL), survival exceeded 90% throughout the experiment. Strain A-8^T^ demonstrated slightly lower virulence, with larval survival at 1 × 10^8^ CFU/mL decreasing to 50% at 24 h and declining gradually to complete mortality by 120 h. At 1 × 10^7^ CFU/mL, survival declined to 50% by 96 h, while lower concentrations resulted in survival rates exceeding 70%. Strain A-7^T^ showed similar results to A-5^T^, with all larvae succumbing within 24 h at 1 × 10^8^ CFU/mL. At 1 × 10^7^ CFU/mL, survival declined steadily to 70% by 72 h, with no further mortalities observed thereafter. Lower concentrations resulted in survival rates above 70% (Figure 4).

At 37 °C, strain A-5^T^ was similarly virulent as 22 °C, with complete mortality at 1 × 10^8^ CFU/mL within 24 h. At 1 × 10^7^ CFU/mL, survival remained at 100% for 24 h, decreased to 90% at 72 h, and dropped to 70% by 120 h. Minimal mortality was observed at lower concentrations, with survival consistently exceeding 80%. Similarly, strain A-8^T^ had virulence consistent with observations at 22 °C, with complete mortality at 1 × 10^8^ CFU/mL within 24 h. At 1 × 10^7^ CFU/mL, survival declined from 90% at 24 h to 50% at 96 h and 20% by 120 h, whilst at lower concentrations survival consistently exceeded 80% (Figure 4). However, strain A-7^T^ exhibited enhanced virulence compared to 22 °C, with the strain causing complete mortality at 1 × 10^8^ CFU/mL within 24 h. At 1 × 10^7^ CFU/mL, larval survival decreased to 70% at 24 h, 50% at 48 h, and 0% by 120 h. Lower concentrations resulted in survival rates above 70%.

Survival rates in both the unmanipulated group and the group injected with 10 μL of sterile PBS remained above 95% throughout the 120 h observation period at both 22 °C and 37 °C, indicating minimal background mortality and confirming that the injection procedure itself did not significantly affect larval survival. In contrast, larvae injected with *V. anguillarum* strain ATCC 43305, used as a positive control, exhibited 100% mortality at all tested concentrations within 24 h at both temperatures.

## 4. Discussion

The genus *Aeromonas* contains Gram-negative, oxidase-positive, and facultative anaerobic bacteria found commonly in aquatic environments, food products, and the microbiota of aquatic organisms. This study sought to characterize three novel isolates obtained from rainbow trout and mussels as part of routine health screening activities. Collectively, the phenotypic and genomic analyses indicated these strains formed novel taxa that we propose to be *Aeromonas ichthyocola* sp. nov. A-5ᵀ (LMG 33534ᵀ = DSM 117488ᵀ), *Aeromonas mytilicola* sp. nov. A-7ᵀ (LMG 33536ᵀ = DSM 117490ᵀ), and *Aeromonas mytilicola* subsp. *aquatica* subsp. nov. A-8ᵀ (LMG 33537ᵀ = DSM 117493ᵀ) (Table 2).

In this present study, a polyphasic approach was taken to characterize the three strains, A-5ᵀ, A-7ᵀ, and A-8ᵀ. Each strain exhibited characteristics consistent with placement within the genus *Aeromonas*, including the primary phenotypic identification tests, cellular morphology, *16S rRNA* sequences, MLPA, genome sequences and ecological distribution and habitat preference predictions. Unsurprisingly, the *16S rRNA* gene was unable to resolve the strains to species level, as there is high interspecies similarity in the *Aeromonas* genus (96.7–100%) [40]. Indeed, this is where the MLPA and genome analyses provided the resolution necessary to propose the new taxa [41,42]. Notably, multilocus sequence analysis (MLSA) and MLPA, which rely on the concatenation of sequences from five or more housekeeping genes, are acceptable to the ad hoc committee for the re-evaluation of bacterial species definitions as robust tools for species delineation [43,44].

Our genomic analyses by ANIb revealed identities of 94.29%, 95.27%, and 95.73% for strains A-5^T^, A-7^T^, and A-8^T^, respectively, whilst ANIm values ranged 95.11%, 95.68%, and 95.95% for these strains. Importantly, the dDDH values for strains A-5^T^, A-7^T^, and A-8^T^ ranged from 56.8─65.9%, which is below the 70% threshold for species delineation [45] and indicates that the strains described in this study likely represent new *Aeromonas* species. In 2020, a comprehensive taxonomic update of the *Aeromonas* genus was published [19], and between 2000 and 2023 several new species were described, such as *A. lusitana* [46], *A. aquatica*, *A. finlandiensis*, *A. lacus* [47], and *A. rivipollensis* [48]. Since 2023, no new *Aeromonas* species have been proposed.

Species in the genus *Aeromonas* are broadly distributed across various ecosystems, particularly aquatic environments [12], and these bacteria have been isolated commonly from surface water, groundwater, drinking water, seawater, and irrigation systems [8,12]. Certain *Aeromonas* spp. are opportunistic pathogens and they have been recovered from human fecal, blood, and wound samples, as well as diseased aquatic hosts, which reflects their propensity to cause infections [8,12]. Consistent with this distribution, strains A-5^T^, A-7^T^, and A-8^T^ were identified in both environmental habitats (soil, rhizosphere, freshwater, and marine systems) and human-associated niches (skin, gastrointestinal tract, oral cavity, and vaginal tract).

Although in silico analysis classified strains A-5^T^, A-7^T^, and A-8^T^ as non-human pathogens, the identification of homologous virulence-associated sequences in their genomes suggests potential pathogenic relevance in aquatic hosts. Homology with known virulence genes from *A. hydrophila* and *A. salmonicida*, both of which cause hemorrhagic septicemia in fish and other aquatic organisms, indicates that our newly described strains may harbor pathogenic potential [49].

Interestingly, the strains A-5^T^, A-7^T^, and A-8^T^ were capable of causing lethality in a dose-dependent manner in *G. mellonella*, an alternative host that has been used to study the virulence of *Aeromonas* species and other aquatic pathogens previously [14,15,50]. *G. mellonella* larvae offer several technical advantages. Their relatively large size, with last instar larvae reaching approximately 2 cm in length and weighing around 250 mg, allows for the precise injection of defined bacterial doses in challenges. Additionally, they can be reared at a range of temperatures (10 °C to 37 °C). In our study, 22 °C was selected for because it represents a relevant temperature where *Aeromonas* species naturally persist in aquatic ecosystems and could cause opportunistic infections in aquatic hosts. Meanwhile, 37 °C was chosen to assess virulence potential towards mammalian hosts. Thus, whilst these findings indicate that strains A-5^T^, A-7^T^, and A-8^T^ may be capable of causing opportunistic infections, further investigations would be necessary to confirm whether they are indeed pathogens, including studies in aquatic hosts. It is intriguing that strain A-7^T^ exhibited greater virulence at 37 °C, perhaps indicating an adaptive advantage under conditions mimicking mammalian hosts, and temperature is known to influence the pathogenicity of *Aeromonas* strains [51]. *G. mellonella* may be a suitable alternative host that may be applied in investigations seeking to unravel the molecular mechanisms underlying the temperature- and strain-dependent differences in virulence. Still, further research is needed to determine the ecological roles and pathogenic potential of the newly described strains, particularly to explore fully their relevance for impacts on aquaculture and public health.

Most *Aeromonas* species are biofilm producers, but we observed that strains A-5^T^, A-7^T^, and A-8^T^ did not form biofilms, which may be due to non-optimal conditions employed in the assay. Indeed, Aksentijevic et al. (2024) [52] reported that *A. salmonicida* isolates produced biofilms at 8 °C but not at 25 °C, indicating the importance of incubation temperature in this process. Moreover, biofilm formation can vary by strain within the same *Aeromonas* species, which suggests genetics plays an important role as well [53]. Based on these findings, future studies will further evaluate the biofilm-forming capacities of the newly identified isolates under different conditions, including temperatures.

Additionally, the identification of prophage elements within the genomes of A-5^T^, A-7^T^, and A-8^T^ is consistent with previous studies demonstrating that *Aeromonas* species frequently harbor mobile genetic elements, including prophages, which contribute to genetic variability and potential virulence. Prophages play a crucial role in the horizontal transfer of genes associated with antimicrobial resistance (AMR) and virulence factors, acting as reservoirs for genetic material that enhances bacterial adaptability [54]. Notably, the presence of PHAGE_Klebsi_ST15_OXA48phi14.1 in A-8^T^ suggests a potential link to horizontal gene transfer events, as this phage is known to be associated with clinically relevant AMR genes [55]. Similarly, the PHAGE_Entero_Mu-related element found in A-7^T^ has been previously implicated in the transfer of virulence-associated genes in *Enterobacteriaceae*, raising questions about its functional role in *Aeromonas* [56]. While these prophage regions do not necessarily indicate direct virulence, their presence underscores the genetic plasticity of these strains and their potential to acquire adaptive traits that may impact their ecological fitness and pathogenicity.

The absence of plasmids in *Aeromonas* strains A-5^T^ and A-8^T^ aligns with previous findings that not all *Aeromonas* strains harbor plasmids, as plasmid carriage rates can be low in this genus [57]. This could suggest that the strains rely solely on chromosomal genes for survival and adaptation, or they may possess genetic factors that prevent plasmid acquisition [57], although loss during culture cannot be ruled out. In contrast, the plasmids identified in strain A-7^T^ encode key proteins involved in mobilization, replication, stability, and potential gene exchange, thus contributing to bacterial adaptation and survival. The presence of MobV and MobQ family relaxases suggests an active role in plasmid mobilization, facilitating horizontal gene transfer between bacterial cells, a process well-documented as a major driver of bacterial evolution and adaptation [58,59]. The plasmid replication protein RepB plays a crucial role in ensuring stable plasmid inheritance and maintenance, which is essential for long-term persistence within bacterial populations [60,61]. Furthermore, the TraY domain-containing protein, known to regulate conjugative transfer, indicates that the plasmid may enhance genetic exchange capabilities, further promoting bacterial adaptability [62]. Additionally, the Type II toxin-antitoxin system RelE/ParE family toxin enhances plasmid stability by selectively eliminating plasmid-free cells [63], thereby ensuring its propagation within the bacterial population, a mechanism widely recognized to be a key factor in maintaining plasmid prevalence, particularly in antibiotic resistance and virulence-associated plasmids [61]. Collectively, these findings indicate that the plasmid content of strain A-7^T^ may play a crucial role in genetic exchange, adaptation, and survival within its environment, aligning with previous studies that highlight the significance of plasmids in bacterial evolution and pathogenicity [62]. The discovery of multiple biosynthetic gene clusters, particularly those linked to ribosomally synthesized and post-translationally modified peptides (RiPPs) and non-ribosomal peptide synthetases (NRPS), further supports the notion that *Aeromonas* species are metabolically versatile bacteria capable of synthesizing bioactive compounds [64]. The consistent presence of amonabactin-associated NRPS clusters in all three strains suggests a strong selective pressure for iron acquisition mechanisms, which may enhance survival in iron-limited environments [65]. Amonabactin, a siderophore, has been well documented in *A. hydrophila*, where it plays a critical role in iron sequestration, promoting bacterial fitness during the host immune response [66]. The presence of these clusters in A-5^T^, A-7^T^, and A-8^T^ suggests that these strains may exhibit similar survival strategies, potentially influencing their ability to colonize diverse ecosystems.

Finally, the identification of antibiotic resistance genes in strains A-5^T^, A-7^T^, and A-8^T^ further supports the role of *Aeromonas* species as reservoirs of clinically significant β-lactamases. While *blaMOX*-9, a widely distributed CMY-1/MOX family β-lactamase [67], was absent in our strains, we detected *TRU*-1 in strain A-8^T^, a β-lactamase associated with β-lactam resistance. Additionally, strain A-7^T^ carried *OXA*-917, a carbapenem-hydrolyzing class D β-lactamase (CHDL), reinforcing the involvement of *Aeromonas* in carbapenem resistance [68]. The detection of *sul2* in strain A-5^T^ highlights *Aeromonas* contribution to multidrug resistance, consistent with previous findings. Efflux pumps, such as MexB in *A. media*, have been linked to the transport of resistance determinants, including *blaMOX*-9 and *blaOXA*-427 [69]. Although our study did not focus on efflux pumps, their role in transporting resistance determinants—such as *blaMOX*-9 and *blaOXA*-427 in *A. media*—suggests that *Aeromonas* employs both intrinsic and acquired resistance mechanisms against antimicrobials. Given the clinical importance of *blaKPC*-1 in multidrug-resistant infections with high mortality rates [70], ongoing surveillance of *Aeromonas* spp. is crucial to understanding their role in antimicrobial resistance dissemination.

## 5. Conclusions

This study identified and characterized three novel *Aeromonas* strains—*Aeromonas ichthyocola* sp. nov. A-5ᵀ (LMG 33534ᵀ = DSM 117488ᵀ), *Aeromonas mytilicola* sp. nov. A-7ᵀ (LMG 33536ᵀ = DSM 117490ᵀ), and *Aeromonas mytilicola* subsp. *aquatica* subsp. nov. A-8ᵀ (LMG 33537ᵀ = DSM 117493ᵀ)—through genomic, phenotypic, and pathogenicity analyses. Genomic approaches, including MLPA, dDDH, and ANI, confirmed their taxonomic novelty and genetic divergence from closely related species. Overall, these findings expand our understanding of *Aeromonas* diversity and demonstrate the utility of genome-based methods for species delineation in this genus.

## Figures and Tables

**Table 1:** Selected physiological and biochemical characteristics of three novel *Aeromonas* strains. Note, results in this table only show differential observations between the strains. +, positive; -, negative; W, weak; ND, not defined; 1: *A. ichthyocola* A-5^T^; 2: *A. mytilicola* A-7^T^; 3: *A. mytilicola* subsp. *aquatica* A-8^T^; 4: *A. media* CECT 4232^T^; 5: *A. hydrophila* ATCC 7966^T^; 6: *A. rivipollensis* P2G1^T^. A comprehensive list of phenotypic traits for each strain is provided in Table S1.

	1	2	3	4 *	5 **	6 ***
Hydrolysis of						
DNA	-	-	W	+	+	ND
Tween 80	+	-	+	+	+	ND
Starch	+	-	+	+	+	ND
L-Tyrosin	-	+	-	-	ND	ND
API 20 NE						
L-Arabinose	+	+	+	-	+	-
API 20 E						
Tryptophan Deaminase	+	-	-	-	-	-
BIOLOG GENIII						
Dextrin	-	+	+	ND	ND	ND
α-D-Lactose	-	+	-	+	-	ND
D-Galacturonic Acid	-	-	+	+	ND	ND
D-Arabitol	-	-	+	-	ND	ND
D-Cellobiose	-	+	+	+	-	ND
Glycerol	-	-	+	+	+	ND
Sucrose	+	+	+	-	+	+
Chemical sensitivity assays						
1% Sodium Lactate	-	+	+	ND	ND	ND
D-serine	-	-	+	ND	+	ND

* Data from Allen et al., 1983 [37]; ** Data from Samayanpaulraj et al., 2020 [38]; *** Data from Reimer et al., 2022 [39].

**Table 2:** Descriptions of *A. ichthyocola*, *A. mytilicola*, and *A. mytilicola* subsp. *aquatica*.

Guiding Code for Nomenclature	ICPN	ICPN	ICPN
Nature of the type material	Strain	Strain	Strain
Genus name	*Aeromonas*	*Aeromonas*	*Aeromonas*
Species name	*Aeromonas ichthyocola*	*Aeromonas mytilicola*	*Aeromonas mytilicola*
Specific epithet	*ichthyocola*	*mytilicola*	*mytilicola* subsp. *aquatica*
Species status	sp. nov.	sp. nov.	subsp. nov
Species etymology	ich.thy.o’co.la. Gr. masc. n. *ichthys*, fish; L. masc./fem. n. suff. -*cola*, dweller; from L. masc./fem. n. *incola*, dweller; N.L. fem. n. *ichthyocola*, a dweller of fish	my.ti.li’co.la. L. masc. n. *mytilus*, a mussel; L. masc./fem. n. suff. -*cola*, dweller; from L. masc./fem. n. *incola*, dweller; N.L. fem. n. *mytilicola*, a dweller of mussels	a.qua’ti.ca. L. fem. n. *aquatica*, pertaining to water, aquatic
Description of the new taxon and diagnostic traits	Cells are Gram-stain-negative, non-motile, aerobic, and rod-shaped, 0.9–1.0 µm long and 1.5–1.6 µm wide. Colonies on Tryptic Soy Agar are convex, smooth, and cream-colored. Oxidase and catalase activities are positive, while indole and H₂S production are negative. Growth occurs on Nutrient Agar, R2A Agar, McConkey Agar, Sea Water Agar, Brain Heart Infusion Agar (BHIA), Tryptic Soy Agar (TSA), and Marine Agar. Growth is also observed on 5% Sheep Blood Agar and Thiosulfate-Citrate-Bile Salts-Sucrose Agar (TCBS). However, no growth is observed on Bile Aesculin Agar. Growth occurs in the range of 4–42 °C, with tolerance to 0–6% NaCl. The strain shows negative hydrolysis for DNA but positive hydrolysis for Tween 20, Tween 80, starch, gelatin, and casein. L-tyrosine hydrolysis is negative. In the API 20NE test, the strain was positive for the reduction of nitrate to nitrite, indole production, fermentation of D-glucose, arginine dihydrolase, hydrolysis of aesculin, hydrolysis of gelatin, and β-galactosidase, but negative for urease. The strain assimilates D-glucose, L-arabinose, D-mannose, D-mannitol, N-acetyl-D-glucosamine, potassium gluconate, malic acid, and sucrose but does not assimilate adipic acid, trisodium citrate, or phenylacetic acid. Biolog GEN III system analysis indicates the strain is positive for the utilization of α-D-glucose, D-mannose, D-mannitol, dextrin, trehalose, and β-methyl-D-glucoside but negative for D-raffinose, D-galactose, D-cellobiose, sorbitol, gelatin, pectin, and Tween 40. Chemical sensitivity assays show the strain is resistant to vancomycin, rifamycin SV, tetrazolium violet, tetrazolium blue, and niaproof 4 but sensitive to nalidixic acid, aztreonam, and potassium tellurite. It can tolerate 1% NaCl, 4% NaCl, and pH 6 but is not tolerant to 8% NaCl or pH 5.	Cells are Gram-stain-negative, motile, aerobic, and rod-shaped, 0.7–0.8 µm long and 1.4–1.5 µm wide. Colonies on Tryptic Soy Agar (TSA) are convex, smooth, and cream-colored. Oxidase and catalase activities are positive, while H₂S production is negative. Growth occurs on Nutrient Agar, R2A Agar, McConkey Agar, Sea Water Agar, Brain Heart Infusion Agar (BHIA), TSA, and Marine Agar. Growth is also observed on 5% Sheep Blood Agar with γ-hemolysis and Thiosulfate-Citrate-Bile Salts-Sucrose Agar (TCBS). However, no growth is observed on Bile Aesculin Agar. The growth temperature range is 10–42 °C, with tolerance to 0–4% NaCl. The strain shows weakly positive hydrolysis for Tween 20 and casein but negative hydrolysis for DNA, Tween 80, and starch. Gelatin hydrolysis is positive, while L-tyrosine hydrolysis is also positive. In the API 20NE test, the strain is positive for the reduction of nitrate to nitrite, indole production, fermentation of D-glucose, arginine dihydrolase, hydrolysis of aesculin, hydrolysis of gelatin, and β-galactosidase. It is negative for urease. The strain assimilates D-glucose, L-arabinose, D-mannose, D-mannitol, N-acetyl-D-glucosamine, and D-maltose but does not assimilate adipic acid, trisodium citrate, or phenylacetic acid. Biolog GEN III system analysis indicates the strain utilizes α-D-glucose, D-mannose, D-mannitol, dextrin, α-D-lactose, trehalose, β-methyl-D-glucoside, D-galactose, and D-cellobiose. It does not utilize D-raffinose, gelatin, or Tween 40. Chemical sensitivity assays show the strain is resistant to vancomycin, rifamycin SV, tetrazolium violet, tetrazolium blue, and niaproof 4 but sensitive to nalidixic acid, aztreonam, and potassium tellurite. It can tolerate 1% NaCl, weakly tolerate 4% NaCl, and survive at pH 6. It cannot tolerate 8% NaCl or pH 5 but tolerates guanidine HCl and 1% sodium lactate.	Cells are Gram-stain-negative, motile, aerobic, and rod-shaped, 0.7–0.9 µm long and 1.6–1.7 µm wide. Colonies grown on Tryptic Soy Agar (TSA) are convex, smooth, and cream-colored. Oxidase and catalase activities are positive, while H₂S production is negative. Growth occurs on Nutrient Agar, R2A Agar, McConkey Agar, Sea Water Agar, Brain Heart Infusion Agar (BHIA), TSA, and Marine Agar. Growth is also observed on 5% Sheep Blood Agar with α-hemolysis and the strain shows weak growth on Thiosulfate-Citrate-Bile Salts-Sucrose Agar (TCBS). However, no growth is observed on Bile Aesculin Agar. The growth temperature range is 4–42 °C, with tolerance to 0–4% NaCl. The strain shows weakly positive hydrolysis for DNA and positive hydrolysis for Tween 20, Tween 80, starch, gelatin, and casein. However, L-tyrosine hydrolysis is negative. In the API 20NE test, the strain is positive for the reduction of nitrate to nitrite, indole production, fermentation of D-glucose, arginine dihydrolase, hydrolysis of aesculin, hydrolysis of gelatin, and β-galactosidase. It is negative for urease activity. The strain assimilates D-glucose, L-arabinose, D-mannose, D-mannitol, N-acetyl-D-glucosamine, potassium gluconate, D-maltose, capric acid, and malic acid. However, it does not assimilate adipic acid or phenylacetic acid. Biolog GEN III system analysis indicates the strain utilizes α-D-glucose, D-mannose, D-mannitol, dextrin, D-cellobiose, and L-malic acid. It does not utilize D-raffinose, D-sorbitol, N-acetyl-β-D-mannosamine, or L-fucose. Chemical sensitivity assays show the strain is resistant to vancomycin, rifamycin SV, tetrazolium violet, tetrazolium blue, and niaproof 4 but sensitive to nalidixic acid, aztreonam, and potassium tellurite. It can tolerate 1% NaCl, weakly tolerate 4% NaCl, and survive at pH 6. It is not tolerant to 8% NaCl or pH 5 but can grow in the presence of D-serine and 1% sodium lactate.
Country of origin	Türkiye	Türkiye	Türkiye
Region of origin	Adapazari	Istanbul	Duzce
Date of isolation (dd/mm/yyyy)	10/01/2015	10/05/2023	28/10/2022
Source of isolation	Rainbow trout (*Oncorhynchus mykiss*)	Mussels *(Mytilus galloprovincialis)*	Rainbow trout (*Oncorhynchus mykiss*)
Sampling date (dd/mm/yyyy)	10/01/2015	12/05/2023	30/10/2022
Latitude (xx°xx′xx″ N/S)	40°40′57.1″ N	41°01′58.5″ N	40°46′20.3″ N
Longitude (xx°xx′xx″ E/W)	30°42′08.5″ E	29°01′24.8″ E	30°58′40.9″ E
16S rRNA gene accession nr.	PQ549964	PQ549960	PQ549963
Genome accession number [RefSeq; EMBL; …]	A-5^T^; JBJSWH000000000	A-7^T^; CP174126	A-8^T^; CP172298
Plasmid accession number [RefSeq; EMBL; …]	-	pA-7: CP174127- CP174129	-
Genome status	Incomplete	Complete	Complete
Genome size	4,676,890	4,736,205	4,681,979
GC %	61.2	61.1	61.4
Number of strains in study	1	1	1
Source of isolation of non-type strains	-	-	-
Information related to the Nagoya Protocol	Türkiye is not yet Party to the Nagoya-Protocol	Türkiye is not yet Party to the Nagoya-Protocol	Türkiye is not yet Party to the Nagoya-Protocol
Designation of the Type Strain	A-5^T^	A-7^T^	A-8^T^
Strain Collection Numbers	LMG 33534; DSM 117488	LMG 33536; DSM 117490	LMG 33537; DSM 117493

## Data Availability

The data presented in this study are available in the article.

## Supplementary Materials

The following supporting information can be downloaded at: https://www.mdpi.com/article/10.3390/ani15070948/s1, Figure S1: Transmission electron micrograph of strains novel *Aeromonas* strains. A-5^T^, A-7^T^, and A-8^T^; Figure S2: Neighbor-joining phylogenetic tree constructed with *16S rRNA* gene sequences showing the phylogenetic positions of strains A-5^T^, A-7^T^, and A-8^T^ among related taxa. Filled circles indicate branch nodes recovered by ML, NJ, and MP phylogenetic trees. Open circles indicate that the corresponding nodes were also recovered in either ML or MP algorithm. The numbers at the branch-nodes represent the percentage of 1000 bootstrap replicates; only values > 70% are depicted in the tree. GenBank accession numbers for *16S rRNA* gene sequences are presented in parentheses. *Escherichia coli* ATCC 11229^T^ was used as an outgroup. Bar, 0.02 substitutions per nucleotide position; Figure S3: Ecological distribution pattern of strains A-5^T^, A-7^T^, and A-8^T^; Table S1: Physiological and biochemical characteristics of novel *Aeromonas* strains. 1: *A. ichthyocola* A-5^T^; 2: *A. mytilicola* A-7^T^; 3: *A. mytilicola* subsp. *aquatica A-8*^T^; Table S2: Genomic characteristics of strains A-5^T^, A-7^T^, and A-8^T^; Table S3: Antimicrobial resistance genes (AMR) genes annotated in genomes of strains A-5^T^, A-7^T^, and A-8^T^; Table S4: Virulence genes annotated in genomes of strains A-5^T^, A-7^T^, and A-8^T^; Table S5: Prophage regions identified in the genomes of strains A-5^T^, A-7^T^, and A-8^T^. Prophage regions were evaluated based on a completeness score: intact (score > 90), questionable (score 70–90), and incomplete (score < 70). These regions include key details such as region length, GC content, and associated phage types.; Table S6: Biosynthetic gene clusters detected in the genome of strains A-5^T^, A-7^T^, and A-8^T^ using the AntiSMASH server; Table S7: Plasmid-associated genes identified in *Aeromonas* strain A-7^T^; Table S8: Prevalence levels of strain across various metagenomes as detected through the IMNGS platform.
