# Supplementary material for: Unraveling Genomic and Pathogenic Features of Aeromonas ichthyocola sp. nov., Aeromonas mytilicola sp. nov., and Aeromonas mytilicola subsp. aquatica subsp. nov
Source: Animals (Basel). 2025 Mar 26;15(7):948. doi: 10.3390/ani15070948 (PMC11988052; doi:10.3390/ani15070948)
Supplement: Supplementary file 1 [file animals-15-00948-s001.zip › Sup. Figure S1-S4 and Sup Tables S1-S7 updated.pdf]

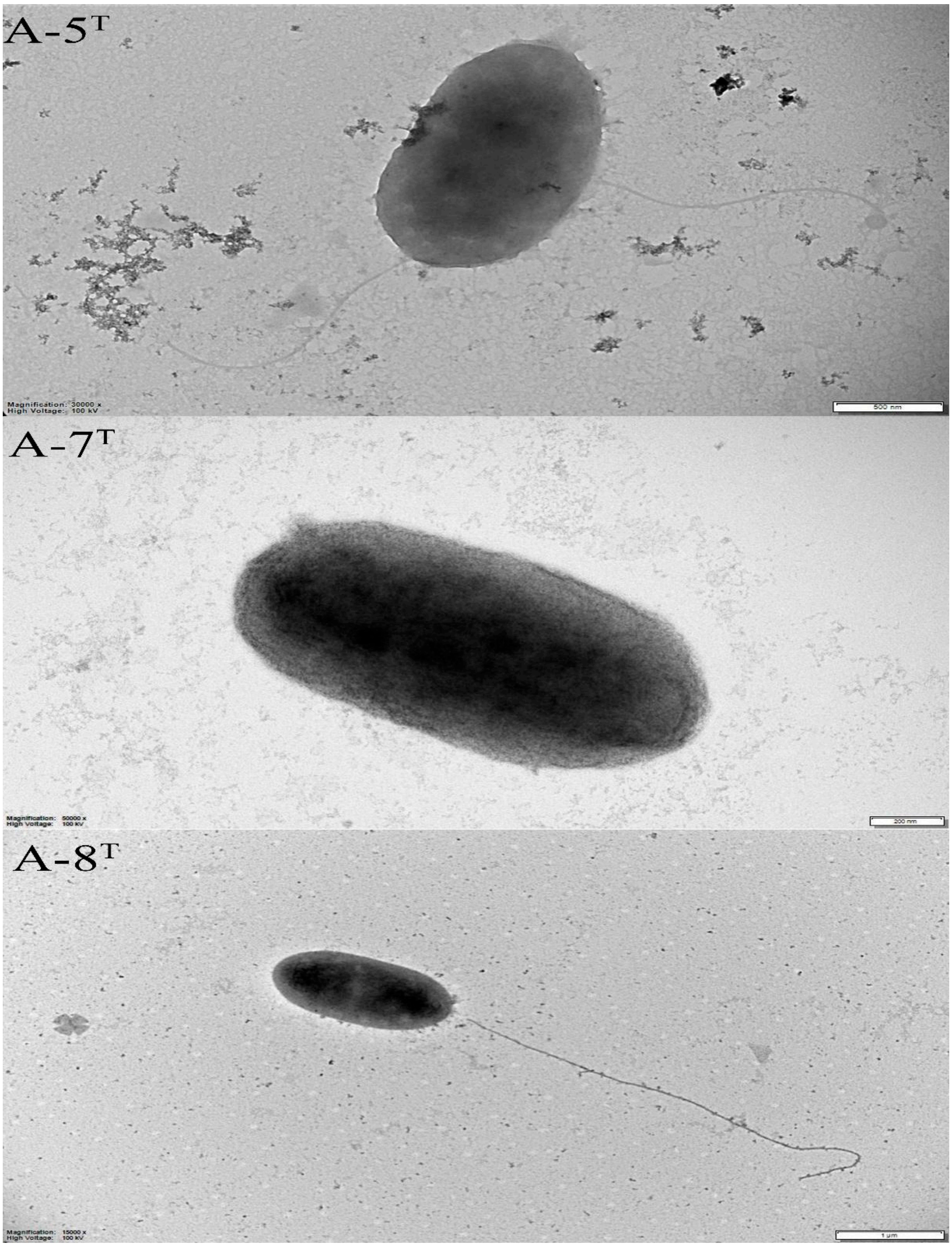

Figure S1: Transmission electron micrographs of strains A-5^T, A-7^T, and A-8^T

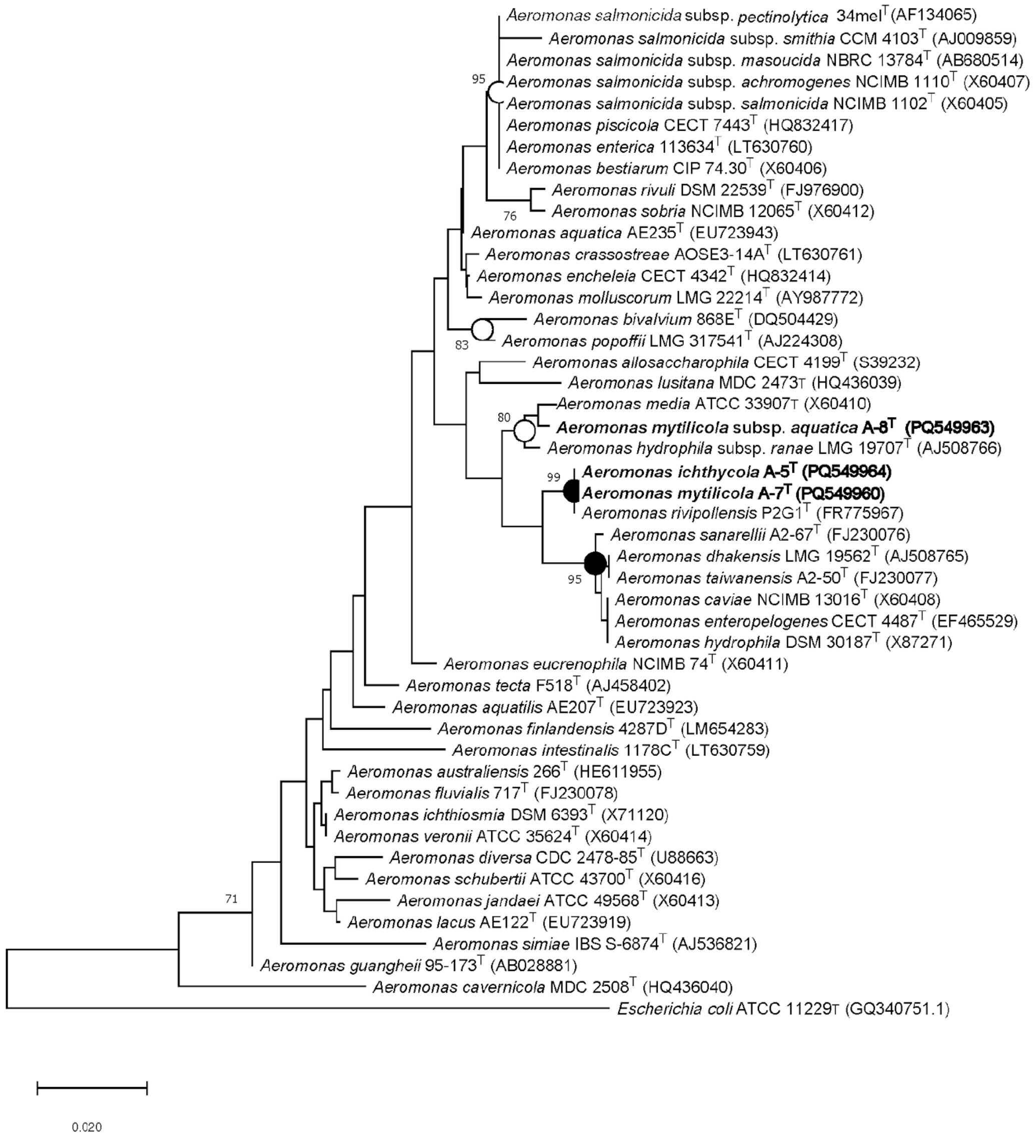

Figure S2: Neighbor-joining phylogenetic tree constructed with *16S rRNA* gene sequences showing the phylogenetic positions of strains A-5^{T}, A-7^{T} and A-8^{T} among related taxa. Filled circles indicate branch nodes recovered by ML, NJ, and MP phylogenetic trees. Open circles indicate that the corresponding nodes were also recovered in either ML or MP algorithm. The numbers at the branch-nodes represent the percentage of 1,000 bootstrap replicates; only values > 70 % are depicted in the tree. GenBank accession numbers for *16S rRNA* gene sequences are presented in parentheses. *Escherichia coli* ATCC 11229^{T} was used as an outgroup. Bar, 0.02 substitutions per nucleotide position

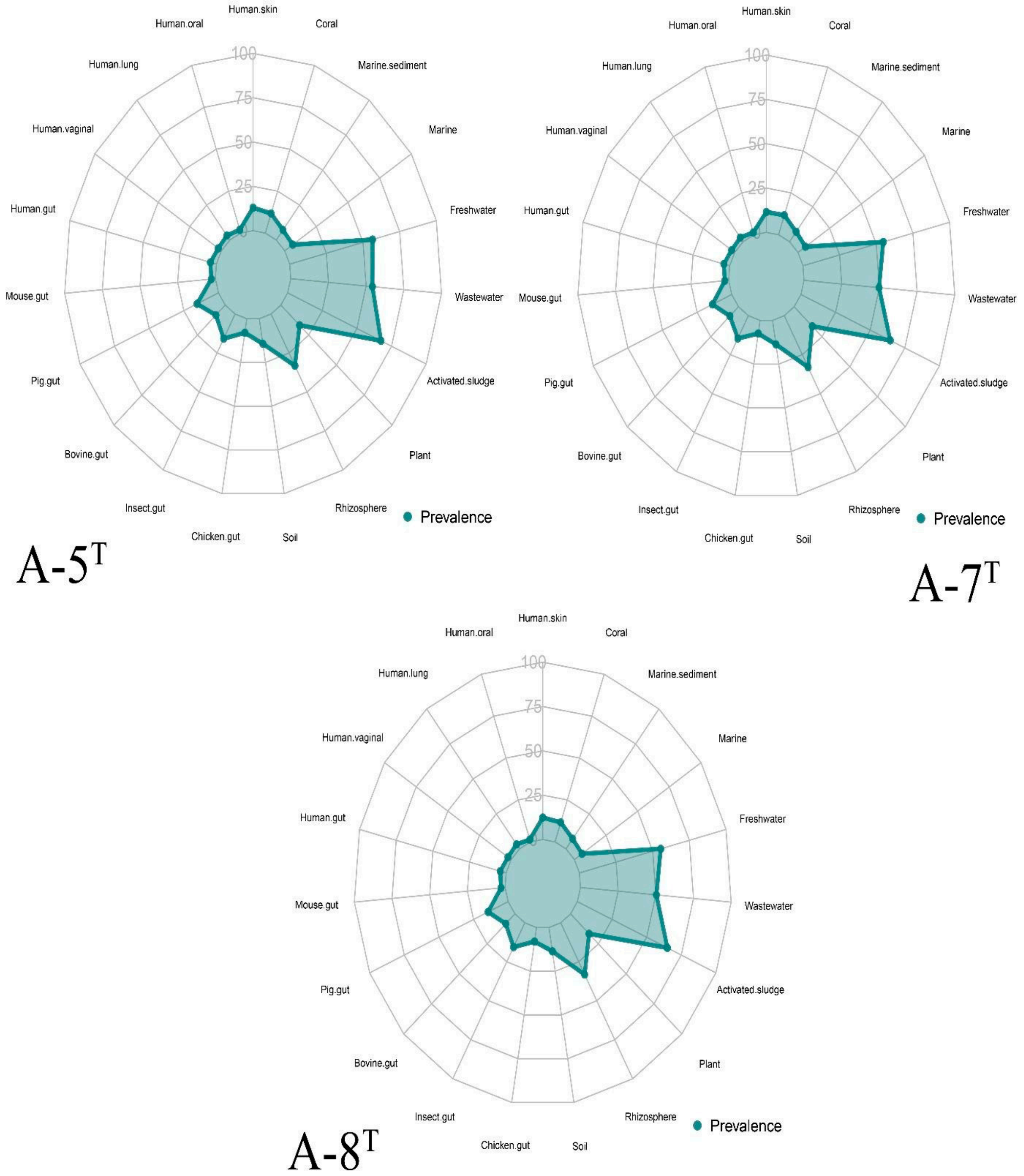

Figure S3: The ecological distribution pattern of the strains A-5^T, A-7^T and A-8^T.

Table S1. Physiological and biochemical characteristics of novel *Aeromonas* strains. **1**: *A. ichthyocola* A-5^T; **2**: *A. mytilicola* A-7^T; **3**: *A. mytilicola* subsp. *aquatica* A-8^T

**Characteristics**	**1**	**2**	**3**
Oxidase	+	+	+
Catalase	+	+	+
Temperature (◦C)	4-42°C	10-42°C	4-42°C
NaCl (%) (w/v)	0-6	0-4	0-4
Motility	+	+	+
**Hydrolysis of**			
DNase	-	-	W
Tween 20	+	W	+
Tween 80	+	-	+
Starch	+	-	+
Gelatin	+	+	+
Casein	+	W	+
L-tyrosin	-	+	-
**Growth ability on**			
Nutrient Agar	+	+	+
R2A	+	+	+
Bile Aesuculin Agar	-	-	-
McConkey Agar	+	+	+
Sea water Agar	+	+	+
Brain Heart Agar	+	+	+
TSA	+	+	+
Thiosulfate-citrate-bile salts-sucrose agar (TCBS)	+	+	-
Marine agar	+	+	+
%5 Sheep Blood Agar	γ	γ	α
Anaerobic environment	+	+	+
**API 20 NE**			
Reduction of Nitrate to Nitrite	+	+	+
Indole Production	+	+	+
Fermentation (D-Glucose)	+	+	+
Arginine Dihydrolase	+	+	+
Urease	-	-	-
Hydrolysis of Aesculin	+	+	+
Hydrolysis of Gelatin	+	+	+
β-galactosidase (Para-NitroPhenyl-ßDGalactopyranosidase)	+	+	+
**Assimilation of:**			
D-Glucose	+	+	+
L-Arabinose	+	+	+
D-Mannose	+	+	+
D-Mannitol	+	+	+

N-acetyl-D-Glucosamine	+	+	+
D-Maltose	+	+	+
Potassium Gluconate	+	+	+
Capric Acid	+	+	+
Adipic Acid	-	-	-
Malic Acid	+	+	+
Trisodium Citrate	-	-	-
Phenylacetic Acid	-	-	-
**API 20 E**			
Ortho-Nitrophenyl-β-galactoside (tests for β-galactosidase activity)	+	+	+
Arginine Dihydrolase	+	+	+
Lysine Decarboxylase	-	-	-
Ornithine Decarboxylase	-	-	-
Citrate Utilization	-	-	-
Hydrogen Sulfide Production	-	-	-
Urease	-	-	-
Tryptophan Deaminase	+	-	-
Indole Production	+	+	+
Voges-Proskauer Test	-	-	-
Gelatinase	+	+	+
Glucose Fermentation	+	+	+
Mannitol Fermentation	+	+	+
Inositol Fermentation	-	-	-
Sorbitol Fermentation	-	-	-
Rhamnose Fermentation	-	-	-
Sucrose Fermentation	+	+	+
Melibiose Fermentation	-	-	-
Amygdalin Fermentation	+	+	+
Arabinose Fermentation	+	+	+
**BIOLOG GENIII**			
**Carbon source utilization assays**			
D-Raffinose	-	-	+
α-D-Glucose	+	+	+
D-Sorbitol	-	-	-
Gelatin	+	+	+
Pectin	+	+	+
p-Hydroxy-Phenylacetic Acid	-	-	-
Tween 40	-	-	-
Dextrin	-	+	+
α-D-Lactose	-	+	-
D-Mannose	+	+	+
D-Mannitol	+	+	+
Glycyl-L-Proline	-	-	-
D-Galacturonic Acid	-	-	+
Methyl Pyruvate	-	+	+
γ-Amino-Butyric Acid	-	-	-

D-Maltose	+	+	+
D-Melibiose	-	-	-
D-Fructose	+	+	+
D-Arabitol	-	-	+
L-Alanine	-	-	-
L-Galactonic Acid Lactone	-	-	-
D-Lactic Acid Methyl Ester	-	-	-
α-Hydroxy Butyric Acid	-	-	-
D-Trehalose	+	+	+
β-Methyl-D-Glucoside	+	+	+
D-Galactose	-	+	+
myo-Inositol	-	-	-
L-Arginine	-	-	-
D-Gluconic Acid	+	+	+
L-Lactic Acid	-	-	-
ß-Hydroxy-D,L-Butyric Acid	-	-	-
D-Cellobiose	-	+	+
D-Salicin	-	-	-
3-Methyl Glucose	-	-	-
Glycerol	-	-	+
L-Aspartic Acid	-	-	+
D-Glucuronic Acid	-	-	-
Citric Acid	-	-	-
α-Keto-Butyric Acid	-	-	-
Gentiobiose	-	-	+
N-Acetyl-D-Glucosamine	+	+	+
D-Fucose	-	-	-
D-Glucose-6-PO4	+	+	+
L-Glutamic Acid	-	-	+
Glucuronamide	-	-	-
α-Keto-Glutaric Acid	-	-	+
Acetoacetic Acid	-	-	+
Sucrose	+	+	+
N-Acetyl-β-D-Mannosamine	-	-	+
L-Fucose	-	-	-
D-Fructose-6-PO4	-	-	-
L-Histidine	-	-	+
Mucic Acid	-	-	-
D-Malic Acid	-	-	-
Propionic acid	-	-	-
D-Turanose	-	-	-
N-Acetyl-D-Galactosamine	-	-	-
L-Rhamnose	-	-	-
D-Aspartic Acid	-	-	-
L-Pyroglutamic Acid	-	-	-
Quinic acid	-	-	-
L-Malic Acid	+	+	+

Acetic Acid	-	-	+
Stachyose	-	-	-
N-Acetyl-Neuraminic Acid	-	-	-
Inosine	-	+	+
D-Serine	-	-	+
L-Serine	+	+	+
D-Saccharic Acid	-	-	-
Bromo-Succinic Acid	-	-	+
Formic Acid	-	-	-
**Chemical sensitivity assays**			
1% NaCl	+	+	+
1% Sodium Lactate	-	+	+
Troleandomycin	-	-	-
Lincomycin	-	-	-
Vancomycin	+	+	+
Nalidixic Acid	-	-	-
Aztreonam	-	-	-
pH 6	+	+	+
4% NaCl	+	W	W
Fusidic Acid	-	-	-
Rifamycin SV	+	+	+
Guanidine HCl	-	+	+
Tetrazolium Violet	+	+	+
Lithium Chloride	-	-	-
Sodium Butyrate	-	-	-
pH 5	-	-	-
8% NaCl	-	-	-
D-serine	-	-	+
Minocycline	-	-	-
Niaproof 4	+	+	+
Tetrazolium Blue	+	+	+
Potassium Tellurite	-	-	-
Sodium Bromate	-	-	-
**Antibiotic susceptibility assays (mm)***			
Florfenicol (30 µg)	12	32	28
Doxycycline (30 µg)	16	26	30
Oxytetracycline (30 µg)	8	30	32
Flumequine (30 µg)	34	40	32
Amoxicillin (25 µg)	0	0	0
Erythromycin (15 µg)	16	20	18
Trimethoprim/sulfamethoxazole (1.25/23.75 µg)	0	22	32
Enrofloxacin (5 µg).	34	36	42
0/129 (2,4-Diamino-6,7-di-iso-propylpteridine phosphate)	0	0	0

+; Positive, **–;** negative, **W;** Weak Positive, ***;** The inhibition zones of the *Escherichia coli* ATCC 25922 strain, used as a quality control strain in the disk diffusion test, were found to be within the reference range specified by the CLSI.

Table S2. Genomic characteristics of strains A-5^T, A-7^T and A-8^T

Characteristics	A-5^T	A-7^T	A-8^T
**GeneBank ID**	JBJSWH000000000<br>0	CP174126-<br>CP174129	CP1722980
**Genome size (bp)**	4,676,890	4,751,464	4,681,979
**Genome Coverage**	274.0	152.0	102
**No. contigs**	18	4	1
**N50 value**	762,425	4,736,205	4,681,979
**GC-content (%)**	61,29	61,14	61,4
**Total genes**	4,511	4,416	4,297
**Protein-coding gene (CDS)**	3,803	4,151	4,090
**rRNA (5S, 16S, 23S)**	8, 6, 7	11, 10, 10	11, 10, 10
**tRNA**	116	124	124
**ncRNA**	7	7	7
**Pseudogenes[a]**	564	103	45

[a]The number of total pseudogenes indicated includes genes with ambiguous residues, frameshifted genes, incomplete genes, genes with internal stops or other multiple problems.

Table S3. Antimicrobial resistance genes (AMR) genes annotated in genomes of strains A-5^T, A-7^T and A-8^T

Strain	AMR gene(s)	Proposed AMR mechanism
**A-5^T**	*APH(4)-Ia, AAC(3)-IVa, APH(6)-Id, APH(3'')-Ib,*	Antibiotic inactivation
	Escherichia coli EF-Tu mutants conferring resistance to Pulvomycin (R234F)	Antibiotic target alteration
	tet(A)	Efflux pump conferring antibiotic resistance
	sul2	Antibiotic target replacement
**A-8^T**	*Escherichia coli EF-Tu mutants conferring resistance to Pulvomycin (R234F)*	Antibiotic target alteration
	TRU-1	Antibiotic inactivation
**A-7^T**	*OXA-917, TRU-1*	Antibiotic inactivation
	Escherichia coli EF-Tu mutants conferring resistance to Pulvomycin (R234F)	Antibiotic target alteration

Table S4. Virulence genes annotated in the genomes of strains A-5^T, A-7^T and A-8^T

Strain	Virulence mechanism	Virulence gene(s)
**A-5^T**	Adherence	*flgC, flgE, flgI, flgJ, fliG, fliP, lafB, lafC, lafE, lafF, lafK, lafS, lafT, lafU, lafX, lfgB, lfgF, lfgG, lfgH, lfgK, lfgL, lfgM, lfhA, lfhB, lfiE, lfiH, lfiJ, lfiM, lfiN, lfiQ, lfiR, maf-5, mshB, mshD, mshE, mshF, mshG, mshI, mshK, mshL, mshM, mshN, mshO, mshP, cheA-2, cheB-2, cheW, cheY, cheZ, flaB, flaJ, flgC, flgE, flgF, flgG, flgH, flgI, flgJ, flgK, flgL, flgM, flgN, flhA, flhB, flhF, flhG, fliA, fliE, fliF, fliG, fliH, fliI, fliJ, fliK, fliL, fliM, fliN, fliO, fliP, fliQ, fliR, flrA, flrB, flrC, maf-1, motX, motY, nueB, pomA2, pomA, pomB2, tapB, tapC, tapD, tapF, tapQ, tapT, tapU, tapV, tapW, tapY1, tppD, tppE, tppF*
	Antiphagocytosis	*wecA*
	Fimbrial adherence determinants	*bcfA, bcfB*
	Immune evasion	*bcfB*
	Secretion system	*exeA, exeC, exeF, exeG, exeI, exeJ, exeL, exeM, exeN, Undetermined, atsC, atsD, atsG, atsH, atsI, atsJ, atsK, atsL, atsP, atsQ, atsS, clpV1, dotU, hcp1, vasH, vasK/atsR, vgrG2, vgrG3, vipA, vipB*
	Stress adaptation	*katG*
	Toxin	*hlyA*
**A-7^T**	Adherence	*mshA, mshB, mshC, mshD, mshE, mshF, mshG, mshI1, mshI, mshJ, mshK, mshL, mshM, mshN, mshO, mshP, mshQ, Undetermined, cheA-2, cheB-2, cheR-3, cheV, cheW, cheY, cheZ, flaA, flaH, flaJ, flgA, flgB, flgC, flgD, flgE, flgF, flgG, flgH, flgI, flgJ, flgK, flgL, flgM, flgN, flhA, flhB, flhF, flhG, fliA, fliE, fliF, fliG, fliH, fliI, fliJ, fliK, fliL, fliM, fliN, fliO, fliP, fliQ, fliR, flmD, flmH, flrA, flrB, flrC, maf-1, maf-2, motX, motY, nueA, nueB, pomA2, pomA, pomB2, pomB, tapB, tapC, tapD, tapF, tapM, tapN, tapO, tapP, tapQ, tapT, tapU, tapV, tapW, tapY1, tppA, tppB, tppC, tppD, tppE, tppF, tppF*
	Secretion system	*exeA, exeB, exeC, exeD, exeE, exeF, exeG, exeH, exeI, exeJ, exeK, exeL, exeM, exeN*
	Stress adaptation	*katG*
	Toxin	*hlyA, rtxA*
**A-8^T**	Adherence	*flgC, flgE, flgI, flgJ, fliF, fliG, fliP, lafB, lafC, lafE, lafF, lafK, lafS, lafT, lafU, lafX, lfgA, lfgB, lfgF, lfgG, lfgH, lfgK, lfgL, lfgM, lfgN, lfhA, lfhB, lfiE, lfiH, lfiI, lfiJ, lfiM, lfiN, lfiQ, lfiR, maf-5, mshA, mshB, mshC, mshD, mshE, mshF, mshG, mshI1, mshI, mshJ, mshK, mshL, mshM, mshN, mshO, mshP, mshQ, cheA-2, cheB-2, cheR-3, cheV, cheW, cheY, cheZ, flaB, flaG, flaH, flaJ, flgA, flgB, flgC, flgD, flgE, flgF, flgG, flgH, flgI, flgJ, flgK, flgL, flgM, flgN, flhA, flhB, flhF, flhG, fliA, fliE, fliF, fliG, fliH, fliI, fliJ, fliK, fliL, fliM, fliN, fliO, fliP, fliQ, fliR, flrA, flrB, flrC, maf-1, motX, motY, pomA2, pomA, pomB2, pomB, tapB,*

		tapC, tapD, tapF, tapM, tapN, tapO, tapP, tapQ, tapT, tapU, tapV, tapW, tapY1, tppA, tppB, tppC, tppD, tppE, tppF, tppF
	Antiphagocytosis	*wecA*
	Fimbrial adherence determinants	*bcfA, bcfB*
	Glycosylation system	*neuB2*
	Immune evasion	*neuB2, rfbA-1*
	Secretion system	*exeA, exeB, exeC, exeD, exeE, exeF, exeG, exeH, exeI, exeJ, exeK, exeL, exeM, exeN, Undetermined, atsC, atsD, atsG, atsH, atsI, atsJ, atsK, atsL, atsP, atsQ, atsS, clpV1, dotU, hcp1, hcp, vasH, vasK/atsR, vgrG1, vgrG2, vgrG3, vipA, vipB*
	Stress adaptation	*katG*
	Toxin	*hlyA, rtxA*

Table S5. Prophage regions identified in the genomes of strains A-5^T, A-7^T and A-8^T . The prophage regions were evaluated based on a completeness score: intact (score > 90), questionable (score 70–90), and incomplete (score < 70). These regions include key details such as region length, GC content, and associated phage types.

Strain	Region	Region Length	Completeness	Score	# Total Proteins	Region Position	Most Common Phage	GC %
**A-5^T**	1	37.7Kb	intact	150	44	305436-343218	PHAGE_Aeromo_phiO18P_NC_009542(31)	58.34%
**A-7^T**	1	35.6Kb	questionable	90	33	2494072-2529681	PHAGE_Escher_Lys12581Vzw_NC_049917(10)	60.70%
**A-7^T**	2	40.1Kb	intact	150	47	3217526-3257643	PHAGE_Entero_Mu_NC_000929(11)	58.70%
**A-8^T**	1	44Kb	intact	150	57	1696976-1741031	PHAGE_Klebsi_ST15_OXA48phi14.1_NC_049454(7)	59.14%

Table S6. Biosynthetic gene clusters detected in the genome of strains A-5^T, A-7^T and A-8^T using the AntiSMASH server

Strain	Region	Type	Start (bp)	End (bp)	Most Similar Gene Cluster	Similarity
**A-5^T**	Region 1.1	RiPP-like	52,581	64,341	-	-
	Region 1.2	NRP-metallophore, NRPS	132,689	186,652	amonabactin P 750	100%
	Region 2.1	RiPP-like	328,397	338,759	-	-
	Region 7.1	hserlactone	166,923	187,549	-	-
**A-7^T**	Region 1.1	terpene-precursor	834,398	855,369	-	-
	Region 1.2	RiPP-like	1,297,789	1,308,631	-	-
	Region 1.3	RiPP-like	2,497,181	2,507,441	-	-
	Region 1.4	NRP-metallophore, NRPS	2,714,017	2,767,994	amonabactin P 750	100%
	Region 1.5	hserlactone	4,210,358	4,230,984	-	-
	Region 1.6	terpene-precursor	3,619,370	3,640,260	-	-
	Region 1.7	hserlactone	4,210,358	4,230,984	-	-
**A-8^T**	Region 1.1	RiPP-like	1,332,604	1,343,446	-	-
	Region 1.2	RiPP-like	2,703,566	2,715,326	-	-
	Region 1.3	NRP-metallophore, NRPS	2,789,795	2,843,821	amonabactin P 750	100%
	Region 1.4	hserlactone	4,149,464	4,170,090	-	-

Table S7. Plasmid-associated genes identified in *Aeromonas* strain A-7^T.

Strain	Accession Number	Locus Tag	Product	Protein ID
A-7^T	CP174127.1	ACIEMD_21985	Replication initiation protein	XKS24857.1
A-7^T	CP174127.1	ACIEMD_21990	Plasmid replication protein RepB	XKS24858.1
A-7^T	CP174127.1	ACIEMD_21995	Mobilization protein	XKS24859.1
A-7^T	CP174127.1	ACIEMD_22000	MobQ family relaxase	XKS24860.1
A-7^T	CP174127.1	ACIEMD_22005	Hypothetical protein	XKS24861.1
A-7^T	CP174127.1	ACIEMD_22010	Hypothetical protein	XKS24862.1
A-7^T	CP174127.1	ACIEMD_22015	Hypothetical protein	XKS24863.1
A-7^T	CP174127.1	ACIEMD_22020	Hypothetical protein	XKS24864.1
A-7^T	CP174127.1	ACIEMD_22025	TraY domain-containing protein	XKS24865.1
A-7^T	CP174127.1	ACIEMD_22030	Type II toxin-antitoxin system RelE/ParE family toxin	XKS24866.1
A-7^T	CP174128.1	ACIEMD_22035	Hypothetical protein	XKS24867.1
A-7^T	CP174128.1	ACIEMD_22040	Plasmid replication protein RepB	XKS24868.1
A-7^T	CP174128.1	ACIEMD_22045	MobV family relaxase	XKS24869.1
A-7^T	CP174128.1	ACIEMD_22050	CopG family ribbon-helix-helix protein	XKS24870.1
A-7^T	CP174128.1	ACIEMD_22055	Type II toxin-antitoxin system RelE/ParE family toxin	XKS24871.1
A-7^T	CP174128.1	ACIEMD_22060	Helix-turn-helix domain-containing protein	XKS24872.1
A-7^T	CP174128.1	ACIEMD_22065	Type II toxin-antitoxin system RelE/ParE family toxin	XKS24873.1
A-7^T	CP174129.1	ACIEMD_22070	Protein Rep	XKS24874.1
A-7^T	CP174129.1	ACIEMD_22075	Hypothetical protein	XKS24875.1
A-7^T	CP174129.1	ACIEMD_22080	Hypothetical protein	XKS24876.1
